# The force-velocity profile as determinant of spike and serve ball speed in top-level male volleyball players

**DOI:** 10.1371/journal.pone.0249612

**Published:** 2021-04-02

**Authors:** Andrés Baena-Raya, Alberto Soriano-Maldonado, Manuel A. Rodríguez-Pérez, Antonio García-de-Alcaraz, Manuel Ortega-Becerra, Pedro Jiménez-Reyes, Amador García-Ramos

**Affiliations:** 1 Department of Education, Faculty of Education Sciences, University of Almería, Almería, Spain; 2 SPORT Research Group (CTS-1024), CERNEP Research Center, University of Almería, Almería, Spain; 3 Physical Performance & Sports Research Center, Pablo de Olavide University, Seville, Spain; 4 Center for Sport Studies, Rey Juan Carlos University, Madrid, Spain; 5 Department of Physical Education and Sport, Faculty of Sport Science, University of Granada, Granada, Spain; 6 Department of Sports Sciences and Physical Conditioning, Faculty of Education, Universidad Católica de la Santísima Concepción, Concepción, Chile; University of Cassino e Lazio Meridionale, ITALY

## Abstract

Understanding the relationship between mechanical variables derived from actions such as jumping, sprinting, or ballistic bench press throwing and sport-specific performance moves is of scientific and practical interest for strength and conditioning coaches for improving training programs. We examined the association between mechanical variables derived from the force-velocity (FV) profiles of the aforementioned actions and spike and serve ball speeds in elite volleyball players. Twenty-two male elite volleyball players (age: 24.3 ± 4.5 years; height: 1.89 ± 0.06 m; body mass: 86.3 ± 8.6 kg) were tested in two sessions. Squatting, sprinting, and bench press throwing FV profiles were determined in the first session, while spike and serve ball speeds were assessed in the second session. The theoretical maximal force (*F*_*0*_) of vertical jumping, the theoretical maximal velocity of sprinting, and the *F*_*0*_ of bench press throwing in ascending order, were strongly associated (r_s_ range 0.53–0.84; p<0.05) with spike and serve ball speeds. These mechanical variables explained 20%-36% of the variability in spike and serve ball speeds, with a greater influence on the serve speed. These results suggest that assessing jumping, sprinting, and bench press throwing force-velocity profiles might help provide player-specific training programs and optimize performance in these technical-tactical actions in male elite volleyball players.

## Introduction

Volleyball is a team sport that requires players to jump, block shots, perform short and explosive movements, and quickly apply positioning strategies while passing a ball over a net with the ultimate goal of scoring points and prohibiting the other team from doing so [1–3]. Decisive actions in volleyball, such as the spike and serve, are typically high-velocity shots comprised by explosive movements such as a quick running approach, a vertical jump and a ball hit [4, 5], which usually help generate most points during a game [6–9]. As spike and serve performance seem to be strong predictors of the outcome of a volleyball match [6, 10], understanding the physical attributes that determine these actions is of practical interest to enhance them through training programs. the quality of these actions is of practical importance to enhance these attributes through training programs.

The spike and serve share a common motor pattern commonly divided into four phases as follows: approach, arm cocking, arm acceleration, and follow-through [1, 11]; these phases are all interconnected to optimize the transfer of momentum throughout the kinetic chain and accelerate the center of mass from a horizontal plane to a vertical plane [12]. Thus, players need a coordinated movement of hip, trunk, shoulder, elbow, and wrist muscles to efficiently transfer their force to the ball [1, 11, 13]. An efficient kinetic chain during spike and serve induces a high ball speed, which is often used to assess performance in decisive actions [2, 14]. Maximum ball speed generated from the greatest hit capability is associated with lower-body and upper-body powers [4, 10, 15]. The former is comprised of a quick running approach, which commonly contains three steps, and a jump (take-off phase), while the latter is related to arm movements, with a previous shoulder hyperextension, and the ball hit. These patterns are also strongly correlated to spike jump height in male players [12]. Thus, producing a quick horizontal movement, proper jump, and efficient upper-body extremity power influences serve and spike performance.

Although strength and power are two of the most important physical aspects in volleyball [2], especially for jump height, the extent to which they are associated with ball speed is unclear. For instance, Forthomme et al. [10] observed a positive association between the isokinetic force of the internal shoulder rotators and spike speed in high-level male volleyball players. However, Valades et al. [14] revealed that although the general strength of the upper limb (i.e., bench press and pullover one-repetition maximum; one-rep max [1RM]) and power parameters (i.e., medicine ball throws) increased throughout the season in professional female volleyball players, ball speed during spike remained unchanged. Understanding how these physical capacities are associated with spike and serve speeds and to what extent in elite volleyball has crucial and practical implications for enhancing volleyball performance.

A new testing methodology has been recently proposed to provide a more comprehensive evaluation of determinants of muscular performance based on force-velocity (FV) profiling [16]. Assessing the FV relationship of such determinants has been proposed to identify the maximal mechanical capabilities of muscles to produce force, velocity, and power in different ballistic actions, such as vertical jumps [17], sprints [18], or bench press throw [19]. The FV relationship is summarized through the following parameters: a) the theoretical maximal force (*F*_0_), the theoretical maximal velocity (*V*_0_), the theoretical maximal power (*P*_max_), and the slope of the FV relationship (FV slope) [16]. Interestingly, an optimal FV slope (the combination of *F*_*0*_ and *V*_0_) exists for each individual, providing meaningful information to prescribe individualized training programs [20, 21]. Understanding the association between these mechanical parameters (*F*_0_, *V*_0_, and/or power) and technical execution during spike and serve hits may improve performance. Specifically, knowing whether specific FV profile-based training programs would help optimize training prescription and maximize spike and serve velocity in volleyball may be beneficial. To our knowledge, no studies have investigated the association of FV profiling with the spike and serve velocity.

Thus, this study aimed to evaluate the association between mechanical variables derived from the jump, sprint, and bench press throw FV profiling and ball speed performed in spike and serve actions in elite volleyball players. First, we hypothesize that the *F*_*0*_ of vertical jumping, sprinting, and bench press throwing would be more related to performance during spike and serve than other mechanical variables. Second, it is hypothesized that spike and serve hits could be modulated by the mechanical variables of the jump, sprint, and bench press throw FV profiles, especially *F*_*0*_.

## Material and methods

### Study design

A cross-sectional experimental design was used to determine the association between the different mechanical variables of FV profiles (*F*_0_, *V*_0_, *P*_max_) and ball speed during the spike and the serve. Volleyball players conducted two testing sessions, separated by 48 h, during their competitive period (January). FV profiles during squat jumping, sprinting, and bench press throwing were determined in the first testing session. Spike and serve ball speeds were assessed in the second testing session. Players were always verbally encouraged to exert maximal effort. No familiarization session was included because all players presented high expertise with the exercises evaluated in the present study. A standardized warm-up was performed at the beginning of each session including 5 minutes of jogging and 5 minutes of lower-limb dynamic stretching (describe the warm-up), and a specific warm-up (described below) was performed before each specific test.

### Participants

Twenty-two male elite volleyball players from the team who ranked first in the First Spanish Division voluntarily participated in this study (age: 24.3 ± 4.5 years; height: 1.89 ± 0.06 m; body mass: 86.3 ± 8.6 kg). We recruited four setters, four opposites, six middle-blockers, and eight outside-hitter players with a mean of 6.68 ± 4.23 years of experience as professional volleyball players. Nine played in the National Team during the last season. Players did not report physical limitations, health problems, or musculoskeletal injuries that could compromise the performance and provided written informed consent before beginning this study. The University of Almería granted Ethical approval to carry out the study (Ethical Application Ref: UALBIO2019/041) and all procedures were performed in accordance with the Code of Ethics of the World Medical Association and the Declaration of Helsinki.

### Jump FV profile test

The specific warm-up for jumping consisted of five countermovement jumps (CMJs) and three CMJs loaded with 30 kg [20]. Thereafter, participants performed maximal vertical jumps without external loads and then jumps with two external loads ranging from 10 to 70 kg. If the strength of the FV relationship showed an R^2^ < 0.97 when three loads were tested, one or two additional loads were tested to ensure an R^2^ > 0.97. The heaviest external load allowed each participant to jump approximately 12 cm (76.20 ± 6.50 kg) [22]. Loaded jumps were performed using a Smith Machine (Multipower Fitness Line, Peroga, Murcia, Spain). Two valid trials were performed with each load with 2 min and 4–5 min of rest between trials and between loading conditions, respectively [17]. Following previous research, jump height was estimated from the flight time recorded by the OptoJump infrared platform (Microgate, Bolzano, Italy) since it has been suggested as the most reliable procedure to determine jump height during loaded CMJs performed on a Smith Machine [23]. Before each jump, participants were instructed to stand up straight while keeping their hands on the hips for jumps without external loads and on the bar for loaded jumps. Thereafter, they squatted until they touched an elastic band attached to the Smith Machine corresponding to a crouching position of 90°, followed by a maximal vertical jump with the ankles, knees, and hips fully extended. To avoid any influence of knee angles on FV profiling, trials were not considered valid if the player’s buttock did not contact the band. A researcher with expertise in this evaluation supervised all trials to ensure that these requirements were met. Only the trial with the highest jump height for each loading condition was used to determine individual FV profiles. Force and velocity values used to determine individual FV profiles were obtained by Samozino et al.’s simple method [24], which only requires three input variables: jump height, push-off distance, and system mass. The push-off distance was determined as the difference between the length of the extended lower limb, measured from the iliac crest to the tip of the toes during plantar flexion, and the vertical distance from the iliac crest to the knee when the latter is flexed at approximately 90° [24]. FV relationship parameters (*F*_0_, *V*_0_, and *P*_max_) were determined through a linear regression model: F (V) = F–aV, where *F*_0_ represents a force-intercept and *a* is the slope of the FV relationship. The velocity-intercept (*V*_0_ = *F*_0_/*a*) relationship and maximum power (*P*_max_ = *F*_0_·*V*_0_/4) were also calculated. *F*_0_ and *P*_max_ were normalized to body mass.

### Sprinting FV profiling

The specific warm-up for sprinting consisted of 3 progressive sprints of 30 m at 50%, 70%, and 90% of the athletes’ self-perceived maximal speed [20]. Thereafter, participants performed two 30-m sprints with maximal speed separated by 4 min of recovery. The trial with the shortest 30-m time was used to determine individual FV relationships. Velocity-time data were recorded at 46.9 Hz with a Stalker Acceleration Testing System (ATS) II radar device (Model: Stalket ATS II Version 5.0.2.1; Applied Concepts, Dallas, TX, USA), which is reportedly valid and reliable for measuring these variables [25]. The radar device was attached to a tripod located 10 m behind the starting line at a height of 1 m, which corresponds approximately to the height of the participants’ center of mass. Participants initiated the sprint from a crouching position (staggered stance). Velocity-time data were used to calculate individual FV relationships and related parameters (*F*_0_, *V*_0_, and *P*_max_) according to the aforementioned method of Samozino et al. [18]. *F*_*0*_ and *P*_max_ were normalized to body mass.

### Bench press throwing FV profiling

The specific warm-up for bench press throwing consisted of one set of five repetitions performed at the maximum intended velocity with an external load of 14 kg (mass of an unloaded Smith machine barbell). To determine individual FV relationships, participants performed 2–3 repetitions with 3 external loads (14 kg, 30 kg, and 50 kg) that were implemented in incremental order. Whenever bar velocity was higher (>0.02 m/s) in the second repetition than in the first repetition, a third repetition was performed to ensure that players performed the test at the maximum intended velocity. The rest period between same-load repetitions was 10–15 s, and that between different-load repetitions was 3–5 min. Bench press throws were performed using a Smith machine (Multipower Fitness Line, Peroga, Murcia, Spain) and a 5-point body contact position technique (head, upper back, and buttocks firmly on the bench, with both feet flat on the floor). The mechanical brake of the Smith machine was used to hold the barbell parallel to the subjects’ nipples and directly above (1–2 cm) their chest for 2 s [19]. From this static position, subjects were instructed to perform a purely concentric action to throw the barbell as high as possible. Two trained spotters were responsible for catching the barbell during its downward movement. Only the repetition with the highest maximum velocity at each load recorded by a linear position transducer (T-Force System, Ergotech, Murcia, Spain) was considered for FV modeling [19]. Maximum force and velocity values recorded at three loads were used to determine FV relationship parameters (*F*_0_, *V*_0_, and *P*_max_) through a linear regression equation.

### Volleyball spike and serve speed test

Ball speeds during spike and serve were tested following the procedure described by Palao & Valades [2]. The specific warm-up for this speed test consisted of 5 min of low-intensity jogging, joint mobilization exercises for upper and lower limbs, and spike and serve technique exercises before finishing with three progressive spikes and serves, exerting at least 95% of the perceived maximal effort of players. Thereafter, each player completed three spikes and serves, each separated by one minute. Tests were held in a daily indoor court (wooden surface). Spike and serves were tested in a randomized order. Participants were instructed to hit the ball as hard as possible within the target area (3×3 m), and the highest ball speed recorded with a Stalker Acceleration Testing System (ATS) II radar device (Stalker ATS II Version 5.0.2.1; Applied Concepts, Dallas, TX, USA) was used for statistical analyses. This device is a valid and reliable instrument for evaluating speed during sprinting and when hitting the ball (r^2^ = 0.99, p < 0.01; ICC 0.96–0.99; CV 0.7–1.9%) compared with photocells [2, 26, 27]. The standard ball used in training and competitions (Molten V5M5000) was used in this study. Players used their normal serve technique during serve performance evaluations. The same trained coach tossed the ball for spike performance evaluation; the ball was thrown 3–4 m upwards at 0.5 m from the net. The hitting area was delimited, and the radar was placed 6 m from the net. The radar recorded ball speed data at 46.9 Hz.

### Statistical analyses

Descriptive data are presented as means and standard deviations. Prior to any statistical analysis, we assessed the distribution of the main variables using the Shapiro–Wilk test. In view of the non-normally distributed data, Spearman’s correlation coefficients (*r*) were used to assess the association between the mechanical variables of FV profiles (*F*_0_, *V*_0_, and *P*_max_) and spike and serve speeds. Qualitative interpretations of *r*_*s*_ coefficients were provided as defined by Hopkins et al. [28]: trivial (0.00–0.09), small (0.10–0.29), moderate (0.30–0.49), large (0.50–0.69), very large (0.70–0.89), nearly perfect (0.90–0.99), and perfect (1.00). To provide a more comprehensive assessment of the aforementioned association, a quantile regression (non-parametric) analysis was used, with either spike or serve speed as dependent variables and FV profile-related variables as independent variables in separate models. The reliability of spike and serve speeds was assessed through the standard error of measurement (SEM = standard deviation of the difference between participant trial results divided by √2), which is expressed in relative terms through the coefficient of variation (CV [%] = SEM / Participants’ mean score × 100) and the intraclass correlation coefficient (ICC; model 3.1). Reliability was quantified using a custom spreadsheet [29], while all other statistical analyses were performed using the software package SPSS version 26.0 (SPSS Inc., Chicago, IL, USA). A p-value < 0.05 was considered statistically significant.

## Results

Spike and serve velocity performance showed high stability and reliability (CV ≤ 2.4%, ICC ≥ 0.99, respectively). The *F*_0_ and *P*_max_ obtained from jump FV profiling, the *V*_0_ and *P*_max_ obtained from sprint FV profiling, and the *F*_0_ and *P*_max_ obtained from bench press throw FV profiling showed a positive association with spike ball speed; this association had moderate (*r* = 0.44; *p*<0.05) to very large effects (*r* = 0.81; *p*<0.01). The *F*_0_ obtained during vertical jumping and the *V*_0_ obtained during sprinting showed the strongest correlation with serve speed (very large correlations; *r* ≥ 0.70). The *F*_0_ from the bench press throwing FV profile was the most associated with both spike and serve speeds (large correlations; *r* ≥ 0.53) (Table 1).

**Table 1:** Spearman’s correlations coefficients and confidence interval between the force–velocity parameters (*F*_*0*_, *V*_*0*_, and *P*_*max*_) and the spike and serve speed.

	Vertical F-V Profile			Horizontal F-V Profile			Bench press throw F-V Profile		
	*F*_*0*_	*V*_*0*_	*Pmax*	*F*_*0*_	*V*_*0*_	*Pmax*	*F*_*0*_	*V*_*0*_	*Pmax*
**Spike**	0.81**	-0.33	0.52**	0.24	0.70**	0.56**	0.55**	0.11	0.44*
**95% CI**	(0.63 to 0.90)	(-0,62 to 0.03)	(0.20 to 0.74)	(-0.13 to 0.55)	(0.45 to 0.85)	(0.25 to 0.77)	(0.24 to 0.76)	(-0.26 to 0.45)	(0.09 to 0.69)
**Serve**	0.82**	-0.50	0.39	0.15	0.72**	0.48*	0.53*	0.05	0.38
**95% CI**	(0.65 to 0.91)	(-0.73 to -0.17)	(-0.03 to 0.66)	(-0.22 to 0.48)	(0.49 to 0.86)	(0.14 to 0.72)	(0.21 to 0.75)	(-0.32 to 0.4)	(0.02 to 0.65)

F0, theoretical maximal force; V0, theoretical maximal velocity; Pmax, maximal power output;

*Correlation is significant (p < 0.05).

**Correlation is significant (p < 0.01).

**Fig 1:**
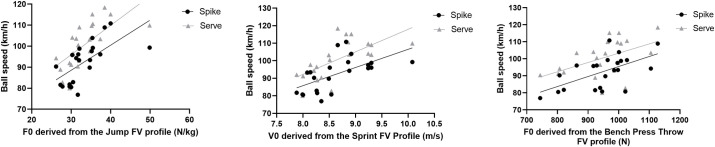
Graphical representation of the association of the mechanical variables derived from jump, sprint and bench press throw force velocity profile with speed of speak and serve in elite volleyball players.

Quantile regression analyses revealed that an additional 1 N·kg^-1^ of force in the *F*_0_ derived from the jumping FV profile was associated with spike ball speed higher by 2.0 km·h^-1^ and a serve speed higher by km·h^-1^ compared to baseline values. An additional 1 m/s of velocity in the *V*_0_ derived from the sprint FV profile was associated with a spike ball speed higher by 10.4 km·h^-1^ and a serve speed higher by 12.8 km·h^-1^ compared to baseline values. Moreover, an additional 1 N·kg^-1^ of force in the *F*_0_ derived from the bench press throwing FV profile was associated with spike ball speed higher by 0.08 km·h^-1^, and a serve ball speed higher by km·h^-1^ compared to baseline values (Table 2). These mechanical variables individually explained 20%-36% of the change in spike and serve ball speed. Fig 1 presents a graphical representation of the association between the main mechanical capabilities generated from FV profile assessments and spike and serve speeds. In all assessments, the influence of mechanical variables was higher on serve ball speed than on spike ball speed.

**Table 2:** Quantile regression analysis examining the association between F_0_ jump, V_0_ sprint and F_0_ bench press throw with the spike and serve speed.

	**Spike Ball Speed**						
	**B**	**SE**	**95% CI**			**P**	**R2**
*F*_*0*_ Vertical	2. 025	0.375	1. 242	-	2. 89	0.001	0.200
*V*_*0*_ Htz	10.4	3854,000	2. 358	-	18. 442	0.14	0.244
*F*_*0*_ BPT	0.079	0.026	0.023	-	0. 134	0.008	0.219
	**Serve Ball Speed**						
	**B**	**SE**	**95% CI**			**P**	**R2**
*F*_*0*_ Vertical	2. 071	0.446	1. 14	-	3,002	0.001	0.280
*V*_*0*_ Htz	12. 857	3. 979	4. 557	-	21,157	0.004	0.361
*F*_*0*_ BPT	0.06	0.0289	-3,157	-	0,120	0.05	0.226

B, unstandardized regression coefficient; SE, standard error; CI, confidence interval; F_0_ Vertical, the theoretical maximal force; V_0_ Htz, the theoretical maximal velocity; BPT, the theoretical maximal force; Spike speed, serve speed.

## Discussion

This study aimed to explore the relationship between jumping, sprinting, and bench press throwing FV profiles and spike and serve ball speeds in male elite volleyball players. The main finding of this study is that the *F*_0_ obtained from vertical jumping, the *V*_0_ obtained from sprinting, and the *F*_0_ obtained from bench press throwing were strongly associated with both spike and serve ball speeds. The abovementioned mechanical variables individually explained 20%-36% of changes in spike and serve ball speeds, which may directly determine the outcome of a volleyball match at elite levels.

In volleyball, vertical jump height, which is linked to the aforementioned running approach, is suggested as a determinant factor to successfully perform spikes and serves; both of these movements are important predictors of attack effectiveness [6]. Although previous studies aimed to maximize vertical jump height by implementing training programs [15, 30], the extent to which vertical jump performance is associated with spike and serve ball speed has received little attention. Specifically, only Challoumas & Artemiou [6] reported a moderate correlation (*r* = 0.52; *p*<0.01) between spike vertical jump height, which is assessed as the highest possible point reached on a vertical jump while mimicking a volleyball spike jump, and ball speed. However, it is known that a single unloaded vertical jump height might not be a good indicator of the athletes’ mechanical output capabilities in comparison to individual FV profiling [31]. The limitations of using isolated jump height measurements are based on several confounding factors that squat jump (SJ) and CMJ tests do not take into consideration, including body mass, push-off distance, the individual FV profile, and optimal FV profile, which directly influence the athletes’ mechanical output capabilities. Conversely, FV profiling addresses this issue by taking these factors into consideration using an accurate computation method, thereby providing meaningful information to individualize jump training programs [21].

Accordingly, the *F*_*0*_ of the jumping FV profile is the most strongly correlated variable with spike and serve ball speeds. In this regard, it may be assumed that a greater ground force application may result in a better transfer of momentum from proximal to distal planes when either spiking or serving [1]. Therefore, these results suggest that hitting performance in volleyball is influenced by the mechanical capacities of lower-body muscles to produce force in a vertical direction. Previous studies reported that *P*_max_ is the most strongly correlated mechanical variable with vertical jump performance [32], suggesting an individualized training program to optimize FV profiling and consequently *P*_max_ [17]. However, from these results, it seems reasonable to suggest that a force-oriented FV profile might be beneficial for elite volleyball players aiming to enhance spike and serve ball speeds. Indeed, the present results suggest that *F*_0_ may explain the 20% and 28% of the variability in spike and serve performance, respectively. It is worthy to note that *F*_0_ is apparently more of a determinant of serving performance rather than of spiking performance as individually tossing the ball might enable players to properly coordinate their movement and effectively apply force, whereas spiking requires timing and temporal coordination between players (setter and hitter) and implies different ball trajectories.

The athlete’s ability to accelerate and reach the highest possible velocity in the shortest period of time may be explained by the sprinting FV profile [33], which provides information on how effectively the athlete applies high levels of force onto the ground at different contraction velocities. This is especially interesting for elite volleyball players who need to accelerate their own body in ballistic push-offs during the quick short running approach to perform a fast tempo attack, or players who need to spike after a previous action, such as a reception or a dig [34]. However, to our knowledge, this is the first study to assess the sprinting FV profile in elite volleyball players. Although an optimal throw technique is the most determining factor of the quality of a force-velocity transfer from the horizontal plane to the vertical plane, the present results suggest that the players’ maximum velocity during sprinting might have a key role in producing high ball speeds during spiking and serving. Accordingly, *V*_0_ is considered to explain 24% and 36% of the variability in spike and serve performance, respectively. Moreover, Wagner et al. [12] stated that angular velocity during the hyperextension movement of the shoulder is crucial for achieving a great spike jump. This argument indicates the need to focus on horizontal velocity during vertical movement and strengthens the association between *V*_*0*_ and ball speed since it is known that vertical force application becomes more important for spike performance as running velocity increases. During a spike jump, the stretch shortening cycle and stiffness play a key role in absorbing and producing force in the shortest time possible, especially in volleyball plyometric actions.

Although upper limb strength and power have been previously assessed in volleyball players, no clear relationships have been reported between them and spike and serve ball speeds [14]. Valades et al. [14] revealed a lack of improvement in spike ball speed in female players throughout an entire season, despite significant increments in the bench press and pull over 1RM. This absence of an association might be because bench press and pull over exercises are not as sport-specific as bench press throwing, which requires maximum acceleration from zero velocity to throw the bar as high as possible in the shortest time [19]. Accordingly, volleyball players are also required to have great arm acceleration directly at the end of the arm cocking phase to hit the ball [1]. Therefore, we selected bench press throwing to assess upper body ballistic performance [19]. As hypothesized, the *F*_0_ of the bench press throwing FV profile was the most correlated mechanical variable with spike and serve ball speeds. Moreover, it was estimated that *F*_0_ may explain 22% and 23% of the variability in spike and serve performance, respectively. Thus, the ability to apply force might be a key performance indicator for positive force transmission to the ball when spiking and serving during the arm acceleration phase. Consequently, it seems reasonable to encourage the assessment of upper body ballistic performance through bench press throwing in elite volleyball players.

Specifically, training programs should focus on enhancing vertical force production through heavy resistance training exercises, such as squats at 80–90% of 1RM [21], improving the maximal velocity during sprinting through assisted countermovement jumps [33] to transfer momentum from the horizontal plane to the vertical plane during the approach phase, and maximizing arm strength and acceleration through specific resistance training exercises, such as bench press throws at 60–80% of 1RM [35] and ballistic movements, such as bench press throwing and medicine ball throws to the floor [14].

Despite the contributions of this study, a few limitations must be addressed. The cross-sectional design of this study inherently established causal relationships. Moreover, although our results suggest that improving specific parameters of jumping, sprinting, and bench press throwing FV profiles might translate into a higher spike and serve ball speed, further prospective and experimental research is needed to confirm or compare these results. As it was difficult to recruit a large sample of elite volleyball players, our sample size was relatively small and future research with larger sample size is warranted. Finally, these results are not generalizable to women or volleyball players with lower performance levels.

## Conclusions

To our knowledge, this study is the first to provide an estimation of the magnitude of the association between different mechanical variables derived from jumping, sprinting, and bench press throwing FV profiles and spike and serve speeds in elite volleyball players. Considering the large number of factors influencing serve and spike performance, these mechanical variables, which approximately explain 20%-36% of the variability in spike and serve speeds, are considered relatively important. Thus, these results will likely help coaches implement specific FV profile-based training programs to improve specific mechanical capabilities that determine spike and serve speeds in elite volleyball players. Moreover, these findings support the need to periodically evaluate FV profiles to make training prescriptions more efficient and beneficial for professional volleyball players.
